# Quality Assessment of Sremska, Nitrite-Free Dry Fermented Sausage Pasteurized with Mild Heat Treatment

**DOI:** 10.3390/foods14193339

**Published:** 2025-09-26

**Authors:** Miroslav Ducic, Jelena Petrovic, Jelena Vranesevic, Danijela Vranic, Milan Baltic, Ljilja Torovic

**Affiliations:** 1 Department of Veterinary Medicine, Faculty of Agriculture, University of Novi Sad, Trg D. Obradovica 8, 21000 Novi Sad, Serbia; miroslav.ducic@polj.uns.ac.rs; 2 Scientific Veterinary Institute Novi Sad, Rumenacki Put 20, 21000 Novi Sad, Serbia; jelena@niv.ns.ac.rs (J.P.); jelenababic@niv.ns.ac.rs (J.V.); 3 Department of Chemical and Physical-Chemical Research, Institute of Meat Hygiene and Technology, Kacanskog 13, 11040 Belgrade, Serbia; danijela.vranic@inmes.rs; 4 Faculty of Veterinary Medicine, University of Belgrade, Bulevar Oslobodjenja 18, 11000 Belgrade, Serbia; baltic@vet.bg.ac.rs; 5 Department of Pharmacy, Faculty of Medicine, University of Novi Sad, Hajduk Veljkova 3, 21000 Novi Sad, Serbia

**Keywords:** dry fermented sausages, sodium nitrite, pasteurization, microbiota, biogenic amines, lipid oxidation, sensory quality

## Abstract

The quality of the Serbian dry fermented sausage, Sremska, was evaluated without added nitrite and pasteurized post-ripening. As an extra safety measure to eliminate *Salmonella*, mild heat treatments (47 °C/6.5 h or 53 °C/22.1 min) were used. The effect of starter culture on product quality was also examined. Sausages were tested at the start of production and after 30 days of storage, measuring physicochemical properties, microbiota populations, biogenic amines, lipid oxidation, and sensory qualities. The absence of nitrite did not cause significant changes in microbiota. Pasteurization lowered total viable counts and lactic acid bacteria by up to 3.5 log CFU/g, especially in sausages with starter culture. *Enterobacteriaceae* were fully eliminated only in pasteurized products. Pathogens like *Listeria monocytogenes* and *Staphylococcus aureus* were not detected. Moderate biogenic amine levels were found in all samples (189.4–312.2 mg/kg), with higher amounts in sausages without nitrite. Neither starter culture nor pasteurization significantly affected biogenic amine levels, although pasteurization helped limit their buildup during storage. Lipid oxidation remained low (0.14–0.25 mg/kg), with slightly higher levels in sausages with starter culture; no changes due to pasteurization or nitrite absence were observed. Sensory evaluation showed all sausages received high scores. Texture, juiciness, aroma, and flavor of sausages made with starter culture were unaffected by nitrite absence or pasteurization. Sausages without starter culture scored slightly lower without nitrite compared to those with it. Pasteurization improved texture, juiciness, aroma plus flavor, and overall acceptability of all sausages without starter culture. Overall, the study concluded that nitrite-free Sremska sausages, when pasteurized with a mild heat process, maintained good quality and enhanced safety.

## 1. Introduction

In the food industry, meat products have traditionally been produced using nitrate/nitrite salts as additives. These artificial ingredients are effective against *Clostridium botulinum* and, to a lesser extent, other bacteria as well. The other effects of nitrite include its contribution to the development of the characteristic cured color and flavor, and its delay of rancidity [1]. However, adding these salts to meat and meat products can lead to the formation of carcinogenic nitrosamine compounds, raising consumer safety concerns [2]. As a result, a new European Commission regulation was introduced to limit the maximum allowed levels of these additives in cured meats. Additionally, the Committee on Environment, Public Health, and Food Safety of the European Parliament recently proposed a motion for a resolution to completely ban added nitrates/nitrites in cured meats within the EU [3]. Over the past decade, consumers have increasingly demanded foods with natural or minimally processed additives, produced through minimally harmful processing technologies—a trend often referred to as the clean label trend [4]. Previous studies have shown that omitting nitrite in DFS is possible if the ripening temperature is lowered, and the production process is prolonged, or if the effect of nitrite is replaced by fruit extract or acid, whereby the quality and safety of such products do not differ from those with nitrite [5,6,7,8]. The use of starter cultures, commercial or autochthonous, helps to achieve desired fermentation and product quality parameters and potentially contributes to the pathogen inhibition in the product [9].

Although microbiologically stable, DFS can pose health risks if raw materials are contaminated with higher levels of foodborne pathogens and/or if antimicrobial controls during manufacturing are insufficient [10]. *Salmonella* is one of the most frequently reported foodborne pathogens, and contamination within the pork production chain remains an ongoing problem in many European countries. In that context, pork-origin DFS have been confirmed as a source of salmonellosis [11,12]. To mitigate this risk, a mild thermal treatment at 47–53 °C after the ripening phase is an effective safety measure, eliminating *Salmonella* without compromising the sensory qualities of the product, as shown by previous research [13]. However, the impact of such heat treatments on other quality aspects, especially in nitrite-free products, requires further assessment. *Clostridium botulinum* presents a biological hazard but does not pose a significant safety concern for DFS, regardless of nitrite presence [14]. However, biogenic amines, mainly produced by microorganisms during fermentation, represent potential biochemical hazards in cured meats [15]. Data on biogenic amines in heat-treated DFS are limited and concern only beef-based products with varying nitrite levels and ripening times [16]. Yet, this study did not examine how applied temperatures affect amine levels during storage. Lipid oxidation, the main cause of deterioration in long-shelf-life meat products, can be exacerbated by heat application, especially in products without nitrite/nitrate salts [17,18]. Regarding the influence of heating on lipid oxidation in DFS, aside from our previous research on cholesterol oxides [19], no peer-reviewed data are available. There are also no published studies on how thermal treatments affect the levels of technological microbiota, such as lactic acid bacteria and coagulase-negative cocci in DFS. In short, previous studies on the application of thermal treatment on DFS were mainly focused on the achieved levels of pathogen reduction [20], while this research investigates, for the first time, how selected temperatures affect certain aspects of the quality of treated products, in addition to the already confirmed antibacterial effect.

Accordingly, this research aimed to evaluate the effects of pasteurization on the quality of pork-based, nitrite-free dry fermented sausage stored for 30 days. Specifically, the impact of mild heat treatments applied post-ripening on microbial populations, lipid oxidation, biogenic amine accumulation, and sensory attributes was investigated. The study also examined how starter culture influences these parameters.

## 2. Materials and Methods

### 2.1. Production of Dry Fermented Sausages

**Figure 1:**
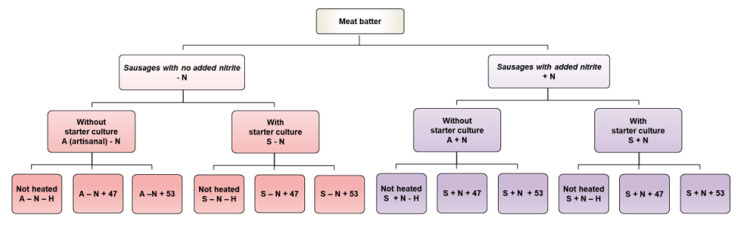
The experimental design. Abbreviations for sausage subgroups: A-N-H: without nitrite, without starter culture, without pasteurization; A-N+47: without nitrite, without starter culture, pasteurized at 47 °C/6.5 h; A-N+53: without nitrite, without starter culture, pasteurized at 53 °C/22.1 min; A+N-H: with nitrite, without starter culture, without pasteurization; A+N+47: with nitrite, without starter culture, pasteurized at 47 °C/6.5 h; A+N+53: with nitrite, without starter culture, pasteurized at 53 °C/22.1 min; S-N-H: without nitrite, with starter culture, without pasteurization; S-N+47: without nitrite, with starter culture, pasteurized at 47 °C/6.5 h; S-N+53: without nitrite, with starter culture, pasteurized at 53 °C/22.1 min; S+N-H: with nitrite, with starter culture, without pasteurization; S+N+47: with nitrite, with starter culture, pasteurized at 47 °C/6.5 h; S+N+53: with nitrite, with starter culture, pasteurized at 53 °C/22.1 min.

A pork-based Serbian DFS (Sremska) was prepared for the study. The experimental design is shown in Figure 1.

*Sausages with no added nitrite.* Sausages were manufactured in a local meat processing plant according to a traditional recipe: pork meat 70%, pork back fat 30%, NaCl 2.5%, spices 2.5% (paprika, chilies, coriander, garlic, and pepper), and dextrose 0.3%. Frozen meat trimmings and fatty tissue were thawed in the refrigerator and then chopped in a bowl cutter into particles about 5 mm in diameter. Subsequently, the other ingredients were added and mixed. For physicochemical and microbiological analyses, samples were taken from the meat batter, which was divided into two batches: one to be produced without and one with the addition of starter culture. A commercial starter culture containing *Lactobacillus sakei*, *Pediococcus pentosaceus*, *Staphylococcus carnosus*, and *Staphylococcus xylosus* (Quick Starter, Lay Gewurze OHG; Grabfeld, Germany) was added at an initial concentration of approximately 6 log CFU/g for each species. Batches were stuffed into industrially prepared natural pork casings (32–34 mm) and placed in a climate chamber for fermentation and drying. Unwanted excessive growth of yeasts and molds on the surface of sausages was suppressed by smoking the product.

*Sausages with added nitrite.* Sausages were prepared from the chopped raw materials in the same proportions and method as described, but with 2.5% of a NaCl-NaNO_2_ mixture (ratio 99.5% NaCl:0.5% NaNO_2_) instead of 2.5% NaCl. The batter was then divided into two batches: one without and the other with added starter culture.

*Ripening of sausages.* The fermentation and drying processes lasted 21 days under the following temperature/humidity conditions: day 1: 20 °C/95%; day 2: 19 °C/90%; day 3: 18 °C/85%; day 4: 17 °C/80%; day 5: 16 °C/75%; days 6–15: 15 °C/75%, and days 16–21: 12 °C/70%. Cold smoking (keeping constant temperature and humidity) was applied daily for 2 h from days 3 to 5.

*Post-processing heat treatment of sausages.* After ripening, a portion of sausages from all batches was analyzed for physicochemical properties before pasteurization and storage. Each of the four batches was then divided into three subgroups: one unpasteurized, one pasteurized at 47 °C for 6.5 h, and the third at 53 °C for 22.1 min. Pasteurization temperatures and durations were selected based on previously determined D-values for *Salmonella* reduction in finished pork DFS, aiming for a 6.5 log CFU/g decrease [13].

*Post-processing and post-pasteurization of sausages.* Both unpasteurized and pasteurized sausages were wrapped in laminated paper sheets simulating retail packaging and stored at 9 °C (the typical household refrigerator temperature in Serbia) [21] for one month. They were then microbiologically and sensorially evaluated and tested for biogenic amines and lipid oxidation.

Three independent production batches of Sremska DFS were prepared for the study.

### 2.2. Microbiological Analysis

From each sausage’s center, 25 g cross sections were added to 225 mL of Maximum Recovery Diluent (MRD; Oxoid, Basingstoke, UK) in filter bags (Nasco, Whirl-Pack 15–23 cm; Fort Atkinson, WI, USA), homogenized with a stomacher for 4 min, and serially diluted (decimal dilutions) in MRD. For lactic acid bacteria counts, dilutions were made in De Man Rogosa Sharpe (MRS) broth. Total viable counts (TVC) were determined using Aerobic Count Plate Petrifilms (3M Health Care, St. Paul, MN, USA), incubated at 30 °C for 72 h. *Enterobacteria* counts (EBC) were performed on *Enterobacteriaceae* Count Plate Petrifilms (3M Health Care, St. Paul, MN, USA) at 37 °C for 24 h. *Escherichia coli* counts (ECC) used *E. coli*/Coliform Count Plate Petrifilms, incubated at 37 °C for 48 h. Lactic acid bacteria (LAB) were counted on Aerobic Count Petrifilms with MRS broth under microaerophilic conditions (Anaerocult C^®^; Merck, Darmstadt, Germany) at 30 °C for 48 h. Coagulase-negative cocci (CNC) were counted on Mannitol Salt Agar (MSA; Oxoid) at 37 °C for 48 h. *Staphylococcus aureus* and *Listeria monocytogenes* were detected using ISO (2021) [22] and ISO (2017) [23] methods, respectively. Microbiological analyses were repeated three times with two technical replicates each (n = 6).

### 2.3. Physicochemical Analysis

Water activity (aw) was measured with a LAB Swift-aw set (Novasina, Schwyz, Switzerland), and pH with a handheld pH meter (Testo 205; Kirchzarten, Germany), following manufacturer guidelines.

### 2.4. Biogenic Amine Analysis

Standards of histamine, tyramine, putrescine, cadaverine, tryptamine, phenylethylamine, spermidine, and spermine were purchased from Sigma Aldrich (St. Louis, MO, USA). Biogenic amines were extracted, purified, and derivatized per the method of Torović et al. [24]. The analysis was performed in three independent experiments with two technical replicates each (n = 6).

### 2.5. Lipid Oxidation Analysis

An index of secondary lipid oxidation products, TBARS (thiobarbituric acid reactive substances) was measured following Tarladgis et al. [25] and Holland [26], calculating malondialdehyde (MDA) levels in mg/kg. Lipid oxidation testing was also performed in triplicate with two replicates each (n = 6).

### 2.6. Sensory Analysis

Sensory evaluation was conducted by a panel of 10 trained food sensory evaluators from the Department of Food Hygiene and Technology at the Faculty of Veterinary Medicine, University of Belgrade. Samples were randomly coded and scored on a 7-point hedonic scale (1 = dislike extremely, 7 = like extremely). Attributes assessed included appearance, cross section appearance, color, texture, juiciness, odor plus flavor, and overall acceptability. All panelists evaluated products in two sessions. The panelists cleaned their palate with plain crackers and cold water between samples.

By national laws, ethical approval for sensory analysis is not required–no human ethics committee or formal documentation process is available. However, the appropriate protocols for protecting the rights and privacy of all participants were utilized during the execution of the research, e.g., no coercion to participate, full disclosure of study requirements and risks, verbal consent of participants, no release of participant data without their knowledge, and the ability to withdraw from the study at any time.

### 2.7. Statistical Analysis

Data from the three replicates were averaged, and one-way ANOVA with Tukey’s post hoc test was used to determine significance, with *p* < 0.05 indicating significant differences, using SPSS software version 30 (SPSS Inc., Chicago, IL, USA). Factorial analysis of variance (ANOVA) and the Pearson correlation test were performed using the STATISTICA version 14.0.015. (TIBCO Software Inc., San Ramon, CA, USA).

## 3. Results and Discussion

### 3.1. Physicochemical Parameters

Physicochemical data were collected at the start (day 1) and end (day 21) of ripening (Table 1).

Initial pH and aw of the meat batter samples ranged from 5.4 to 5.6 and about 0.95, respectively, consistent with previous findings for Sremska sausages at the same stage [27]. After ripening, significantly lower pH values (5.0–5.3) were found in samples produced with starter culture, compared to those without (S-N-H vs. A-N-H; S+N-H vs. A+N-H). The high metabolic activity of the starter culture in the presence of sugar, i.e., lactic acid production, explains this decline, which can improve product safety and stability. However, excessive acidification below pH 5 can produce a strongly acidic and less preferred flavor [28], but all pH values remained above this threshold. Notably, lower pH did not reduce aw, which ranged from 0.71 to 0.76 after ripening, with no significant difference between groups with or without starter culture (*p* > 0.05). Regarding sodium nitrite’s effect, no significant differences in physicochemical parameters were observed between batches with or without this additive (A-N-H vs. A+N-H; S-N-H vs. S+N-H), similar to reports by Perea Sanz et al. [29] and Hospital et al. [30].

### 3.2. Microbiological Parameters

Microbiological analyses evaluated how different factors—presence or absence of sodium nitrite, starter culture, and pasteurization—affected microbiota in samples taken at the start and after 30 days of storage. Results are shown in Table 2.

Initial microbial levels (TVC, EBC, and ECC as hygienic indicators; LAB and CNC) aligned with prior studies [27,31], indicating good hygiene. In these studies, microbiota populations ranged from TVC 5–6.5 log, EBC 3.5–4.5 log, ECC 1–3.5 log, LAB 4–5.5 log, and CNC 3–4 log. Pathogens like *L. monocytogenes* and *S. aureus* were not detected in raw or final products.

After storage, unpasteurized samples without starter culture maintained high TVC levels (8–8.5 log CFU/g), similar to the previous report [27], while those with starter culture (S-N-H; S+N-H) showed lower counts (7.5–8 log CFU/g), likely due to reduced LAB levels (Table 2). The starter culture strains exhibited less stress tolerance during ripening and storage compared to indigenous populations (as reported by Dučić et al. [10] regarding Chorizo de Leon sausages), as seen in the slightly lower LAB counts (8–8.5 vs. 7–7.5 log CFU/g) (Table 2).

Sodium nitrite did not significantly affect total viable or LAB counts in this study (A-N-H vs. A+N-H and S-N-H vs. S+N-H, *p* > 0.05), matching findings by Perea-Sanz et al. [29]. In studies by Tabanelli et al. [5], Christieans et al. [32], and Hospital et al. [33], LAB counts in DFS prepared with starter culture were at comparably higher levels, reaching 8–9 log CFU/g in final products. Nevertheless, results from all these studies show that the absence of nitrite, or its use in low amounts, does not significantly change LAB population levels in DFS products.

After storage, coagulase-negative cocci ranged from 5 to 6 log CFU/g, slightly higher (~0.5 log CFU/g) in starter-inoculated batches (S-N-H vs. A-N-H and S+N-H vs. A+N-H, *p* > 0.05), suggesting these strains adapted well to ripening and storage conditions. The reported range is consistent with previous findings for other types of DFS [30,34].

Similarly, as for LAB, no significant differences were found between CNC levels in DFS subgroups prepared without and with added nitrite (A-N-H vs. A+N-H; S-N-H vs. S+N+H). Conversely, Hospital et al. [33] reported that nitrite, added at the same maximum concentration as in our study (150 mg/kg), reduced CNC numbers by 2 log CFU/g in final products. However, Tabanelli et al. [5], in their study of Italian DFS, observed no decrease in CNC levels with a mix of NaNO_2_ and KNO_3_ at 50 mg/kg and 150 mg/kg, respectively. In these studies and our own, strains of *St. xylosus* and *St. carnosus* served as starter cultures, indicating that nitrite can significantly lower CNC levels in DFS, depending on the susceptibility of the starter strains used.

In the current study, enterobacteria were analyzed as a hygiene indicator of the DFS production process, with *E. coli* specifically indicating fecal contamination. In non-pasteurized DFS (A-N-H; A+N-H; S-N-H; S+N+H), bacterial levels decreased during ripening and after 30 days of storage, reaching 1–2 log CFU/g and falling below detection limits (<1 log CFU/g), respectively. During manufacturing and storage—under standard conditions in the sausage matrix (pH reduction, decreased water activity, anaerobic environment, competitive microbiota)—the growth of enterobacteria was expectedly inhibited, although they persisted at low levels in the final products. The application of mild heat treatment as an additional safety step successfully eliminated all enterobacteria from the pasteurized subgroups (A-N+47 °C; A+N+47 °C; A-N+53 °C; A+N+53 °C; S-N+47 °C; S+N+47 °C; S-N+53 °C; S+N+53 °C). Moreover, this mild heat reduced populations of other microbial groups in DFS (Table 2).

Significant reductions in TVCs and LAB counts were observed across all pasteurized, starter-culture-made DFS subgroups compared to their unpasteurized equivalents. In contrast, in DFS without starter culture, such differences (*p* < 0.05) were only observed in LAB counts in subgroups pasteurized at 47 °C, regardless of nitrite presence, indicating that indigenous LAB populations may have higher thermotolerance than the starter strains of *L. sakei* and *P. pentosaceus* used here. The more acidic pH in starter-culture DFS subgroups might also enhance LAB sensitivity to heat, as microorganisms are generally more vulnerable when the pH is outside their optimal growth range [35]. Despite pasteurization, LAB remained the predominant microbial group in all examined sausages, contributing to both safety and sensory development during storage.

Regarding CNC, all pasteurized sausages showed significant reductions (*p* < 0.05) compared to unpasteurized ones, with larger differences in starter culture subgroups (Table 2). This aligns with the effects observed on LAB and suggests that the starter strains might be less thermotolerant than indigenous microbiota. Therefore, selecting thermotolerant starter strains well adapted to particular DFS types is preferable for products that undergo pasteurization. Despite reductions, CNC levels remained sufficient to support color stability, inhibit rancidity, and enhance sensory qualities by releasing aromatic compounds.

The factorial ANOVA was performed to assess the main effects of nitrite, starter, and pasteurization, as well as their interactions, providing a statistically appropriate evaluation of the factorial design (the experimental design is a 2 × 2 × 3 factorial (Nitrite × Starter × Pasteurization)). The analysis was applied to the microbial counts showing meaningful variation (TVC—total viable count; LAB—lactic acid bacteria; CNC—coagulase-negative cocci, and EBC–enterobacteria count). *E. coli*, *L. monocytogenes*, and *S. aureus* were omitted because their counts were consistently very low/absent, showing minimal differences between treatments. Three-way factorial ANOVA (Table 3) indicated that the main effects of nitrite, starter, and pasteurization were significant for TVC, LAB, and CNC, whereas EBC was only marginally influenced by nitrite (*p* = 0.0517). Several two-way interactions were also significant, demonstrating that the effect of one factor is dependent on the level of another. The three-way interaction reached significance only for TVC (*p* = 0.0435), while it was not significant for the other microbial counts, indicating that the combined influence of all three factors is most pronounced for total viable counts. These findings emphasize the role of both individual factors and their interactions in shaping microbial dynamics.

### 3.3. Biogenic Amines

The eight main biogenic amines (histamine, tyramine, putrescine, cadaverine, tryptamine, phenylethylamine, spermidine, spermine) were analyzed after 30 days of storage (Table 4) in Sremska DFS to assess how different conditions affected their formation.

Only phenylethylamine was below detection in all samples (<0.25 mg/kg). The other seven amines accumulated at moderate levels consistent with data from commercial DFS in Serbia, Portugal, and Belgium [24,36,37], suggesting high-quality raw materials and good hygiene during manufacturing.

The total biogenic amines (288.7–312.2 mg/kg) did not significantly differ between sausages produced with or without starter culture (A-N-H vs. S-N-H; A+N-H vs. S+N-H), indicating that starter strains did not reduce biogenic amine content. Conversely, nitrite addition caused a significant decrease (*p* < 0.05) in total biogenic amines in both sets of sausages compared to those without nitrite (A-N-H vs. A+N-H; S-N-H vs. S+N-H), corroborating findings by Kurt & Zorba [16], who found that higher nitrite levels inhibit the formation of tyramine and cadaverine, likely through antimicrobial effects on proteolytic bacteria. Additionally, putrescine levels were significantly lower (*p* < 0.05) in sausages with added nitrite compared to those without (A+N-H vs. A-N-H; S+N-H vs. S-N-H). Histamine levels were low (4.4 to 19.1 mg/kg) across all samples after one month, and only those with starter culture and nitrite showed a significant decrease (S+N-H vs. S-N-H). This is expected, as only certain strains of bacteria and LAB can produce histamine [38], which are typically not present in high numbers unless specific contamination occurs.

In DFS produced without starter-culture, putrescine was most abundant at 129.3 mg/kg in A-N-H, while tyramine dominated in starter-culture samples at 141.9 mg/kg in S-N+53, consistent with the lower pH observed in these groups, as acidic conditions promote tyramine but reduce putrescine production [39,40].

Heat treatments only lowered putrescine and cadaverine (*p* < 0.05) levels, particularly in starter-culture DFS pasteurized at 53 °C (S-N+53 and S+N+53), likely due to the heat sensitivity of the bacteria producing these amines, especially in more acidic environments.

Tryptamine content remained low, with no significant differences across subgroups, aligning with its classification as a minor amine in fermented sausages, along with absent phenylethylamine [41]. Spermine levels exceeded those of spermidine in all samples, with proportions consistent with previous reports of DFS [16,24,42].

The addition of pasteurization as a safety step to eliminate potential *Salmonella* did not significantly reduce total biogenic amines. Still, pasteurization of finished sausages is useful for preventing the accumulation of biogenic amines. According to EFSA [43], these substances can accumulate during storage and retail, especially in inadequate conditions. Since *Salmonella* are potent producers of biogenic amines [44], pasteurization is a valuable safety measure. Nonetheless, it should supplement, not replace, proper hygiene and manufacturing practices.

To investigate potential relationships between microbiological characteristics and biogenic amine levels in the products, correlation analysis was performed on microbial counts showing meaningful variation (TVC—total viable count; LAB—lactic acid bacteria; CNC—coagulase-negative cocci; and EBC—enterobacteria count) and the corresponding biogenic amine concentrations (Table 5). Counts of *E. coli*, *L. monocytogenes*, and *S. aureus* were excluded from the analysis, as they were consistently very low or undetectable, exhibiting minimal variation across treatments.

Putrescine and spermidine levels were strongly positively correlated with TVC and LAB, while histamine and tyramine showed moderate negative correlations, suggesting that overall microbial growth and lactic acid bacteria, and the formation of specific biogenic amines, could be related. CNC and EBC correlations were generally moderate to weak, indicating a lesser role in biogenic amine accumulation.

### 3.4. Lipid Oxidation

The TBARS test measures secondary lipid oxidation products, particularly malondialdehyde, serving as an indicator of meat quality deterioration from lipid oxidation [17]. The results of the analyses are presented in Table 6.

In all tested sausages after one month of storage, mean TBARS levels ranged from 0.14 mg/kg to 0.25 mg/kg, which aligns with recommended acceptable levels up to 0.3 mg/kg [45], indicating the good quality of the DFS produced in this study. In our research, neither the absence of nitrite nor mild heat treatments affected oxidation as measured by TBARS. Similarly, Sammet et al. [46], in their study of industrially produced fermented sausages, reported that the absence of nitrite did not cause changes in TBARS levels. Regarding sausages prepared without added nitrite and without starter culture, Lorenzo et al. [47] recorded results of 0.78 mg/kg of malondialdehyde, which is higher than in our study. Nonetheless, these authors noted that values under 1 mg/kg do not produce an organoleptic perception of lipid oxidation or any negative impact on the sensory quality of products.

Interestingly, in the current study, the TBARS content was influenced by the presence of the starter culture. Unpasteurized Sremska sausages with added starter (S-N-H; S+N-H) had significantly higher (*p* < 0.05) TBARS levels than their counterparts (A-N-H; A+N-H). These results could relate to higher lipid oxidation activity among the starter culture strains compared to the indigenous microbiota. Different starter culture strains have shown varying levels of lipid oxidation activity in other studies. Van Ba et al. [40], Rodriguez-Gonzales et al. [48], and Oral & Kaban [49] found that LAB and CNC isolates from commercial starter cultures and from the native microbiota of traditional products, respectively, caused higher TBARS levels in experimental sausages than the indigenous microbiota in control samples. Conversely, significant reductions in TBARS levels were observed in products inoculated with strains selected for their ability to limit lipid oxidation in studies by Wang et al. [50] and Sun et al. [51]. Therefore, starter culture strains for meat product production, including DFS, should have limited lipid oxidation activity.

Removing sodium nitrite did not increase TBARS levels in the Sremska sausages in this study. Additionally, mild heat treatments through pasteurization of finished products did not elevate lipid oxidation in DFS. These findings complement our earlier research focusing on cholesterol oxides as another harmful lipid oxidation product [19]. Specifically, in that study, the same pasteurization process was applied to Sremska sausages made without added sodium nitrite. No increase in cholesterol oxidation was observed, and pasteurization actually improved the sausages’ lipid oxidation status [19]. Therefore, our results clearly show that heating up to 53 °C does not negatively impact lipid oxidation in DFS, regardless of nitrite presence.

### 3.5. Sensorial Attributes

Sensory analysis was conducted to assess how Sremska sausages were perceived under the tested conditions (presence/absence of sodium nitrite, starter culture, and pasteurization) after 30 days of storage (Table 7).

The sensory panel rated all sausages made with starter culture very consistently, with high, favorable scores. The absence of nitrite did not cause noticeable changes in color or other sensory attributes. This suggests consistent product quality, likely due to the effect of the starter culture strains used, which support fermentation. In contrast, sausages without starter culture received more variable scores. However, all sausages without starter culture (A-N-H; A-N+47 °C; A-N+53 °C; A+N-H; A+N+47 °C; A+N+53 °C) scored higher in external appearance, cut surface, and color than their starter-inoculated counterparts.

Regarding sodium nitrite’s influence on sensory attributes, subgroups with nitrite (A+N-H; A+N+47; A+N+53) generally received higher scores than nitrite-free sausages (A-N-H; A-N+47; A-N+53). This can be partly explained by the known positive effects of nitrite on meat’s organoleptic qualities, such as color, flavor, and texture [52].

Interestingly, subgroups of sausages without both starter culture and nitrite showed some sensory improvements after pasteurization. Specifically, A-N+47 and A-N+53 scored slightly and significantly higher, respectively, for texture, juiciness, odor plus flavor, and overall acceptability than their unpasteurized equivalent (A-N-H). Moreover, the sensory attributes of these DFS subgroups, improved by mild heat, were rated as good or better than unpasteurized sausages with nitrite (A+N-H). Finally, sausages without starter but with added nitrite and pasteurized at 47 °C (A+N+47) also scored higher than their unpasteurized counterparts (A+N-H). This indicates that mild heat treatment altered the microstructure of the sausage matrix in a way that enhanced overall acceptability and sensory attributes. Lipid melting at 47 °C or 53 °C may also have contributed. However, this melting likely occurred only partially, since lipids reside mainly in fat tissue complexes, which remain stable even when exposed to 75 °C [53], well above our pasteurization temperatures.

No sensory improvements were seen in pasteurized sausages made with starter culture compared to unpasteurized ones, probably because higher acidity causes more protein coagulation, leading to a more stable sausage matrix less affected by mild heat treatment. In this study, the pH of sausages with starter culture was closer to the isoelectric point of meat proteins in DFS (pH 4–5) than that of samples without starter. It is known that at this pH, myofibrils have minimal solubility and water holding capacity [54]. Previous research on Norwegian beef and lamb DFS types by Heir et al. [55] showed that low pasteurization temperatures (43 °C) caused only minor sensory differences between heat-treated and untreated sausages. Interestingly, they reported that sensory scores of heat-treated DFS increased after storage [55].

To investigate the relationship between lipid oxidation and the sensory properties of the product, correlation analysis was performed between TBARS (malondialdehyde) content and sensory attributes. According to the correlation coefficients (Table 8), and given that a negative correlation reflects higher lipid oxidation being associated with lower sensory scores, TBARS values showed weak negative correlations with most sensory traits, particularly surface appearance and color, suggesting that lipid oxidation may slightly compromise visual quality and overall acceptability. In contrast, juiciness exhibited a moderate positive correlation, while odor and flavor appeared largely unaffected.

Overall, all sausage subgroups received favorable scores from the sensory panel, ranging from 5.3 to 7, regarding overall acceptability. These results demonstrate the acceptability of the treated Sremska DFS and suggest good market potential.

## 4. Conclusions

The traditional Serbian dry-fermented sausage, Sremska, was evaluated after storage concerning nitrite presence, starter culture, and pasteurization by mild heat treatment. Microbial populations in all dry-fermented sausage samples, regardless of processing conditions, were at levels that supported product quality. *Enterobacteria* persisted through ripening and storage at low levels in unpasteurized sausages, while pasteurization eliminated them. A moderate level of biogenic amines indicated good product quality, although higher levels appeared in sausages without nitrite than with. Pasteurization limited biogenic amine formation during storage. TBARS levels were low across all samples, and neither nitrite omission nor pasteurization changed TBARS values. Higher TBARS levels were seen in sausages with starter culture, suggesting these strains cause more oxidation. However, their limited activity makes them suitable for production. Neither nitrite omission nor pasteurization affected the sensory perception of dry-fermented sausages made with the starter culture. In samples without a starter, the absence of nitrite slightly lowered acceptability, but mild heat treatment generally improved it. All dry-fermented sausage samples scored well overall.

As most of the previously published works focused on the reduction in pathogens, the novelties in study findings relate to the following: mild heat treatments of nitrite-free dry fermented sausages allow the survival of useful technological microbiota, contributing to the quality of the final product; help limit biogenic amine buildup during storage; do not increase lipid oxidation reactions; and improve certain sensorial attributes. Overall, this study confirms that mild heat treatment of nitrite-free Sremska sausage is a practical step that aligns with consumer demand for safe, minimally processed food free of artificial additives, and should be of interest to both academic researchers and the meat industry.

## Figures and Tables

**Table 1:** Physicochemical characteristics of Sremska sausages. Results are expressed as the mean values ± standard deviation of 6 replicates (n = 3 × 2).

	Meat Batter	Sausages Without Starter Culture *		Sausages with Starter Culture *	
		A-N-H	A+N-H	S-N-H	S+N-H
pH	5.53 ± 0.06	5.69 ± 0.09 a **	5.64 ± 0.11 a	5.23 ± 0.07 b	5.15 ± 0.09 b
Water activity (a_w_)	0.956 ± 0.005	0.730 ± 0.013 a	0.736 ± 0.010 a	0.717 ± 0.012 a	0.737 ± 0.020 a

* Analyses were performed after 21 days of ripening. ** Different letters in a row, but excluding the meat batter values, indicate that values differ significantly (*p* < 0.05). For abbreviations of subgroups of sausages, see Figure 1.

**Table 2:** Counts of microbial groups (log CFU/g) in Sremska sausages. Results are the mean of three independent repetitions and are expressed as mean values ± standard deviation.

Microbial Group	Meat Batter	Sausages After 30 Days of Storage (9 °C)											
		Sausages without Starter						Sausages with Starter					
		Without Added Nitrite			With Added Nitrite			Without Added Nitrite			With Added Nitrite		
		A–N–H	A–N+47	A–N+53	A+N–H	A+N+47	A+N+53	S–N–H ^A^	S–N+47 ^B^	S–N+53 ^C^	S+N–H ^D^	S+N+47 ^E^	S+N+53 ^F^
TVC	6.2 ± 0.3	a * 8.3 ± 0.4 B,C,D,E,F **	a 7.9 ± 0.3 B,C,E,F	a 8.1 ± 0.3 B,C,E,F	a 8.2 ± 0.3 B,C,E,F	a 7.7 ± 0.2 B,C,E,F	a 8.2 ± 0.3 B,C,E,F	*a* *** 7.7 ± 0.4	*b* 6.4 ± 0.3	*c* 5.0 ± 0.4	*a* 7.5 ± 0.3	*c* 5.0 ± 0.5	*d* 4.1 ± 0.6
LAB	5.6 ± 0.2	a 8.5 ± 0.2 A,B,C,D,E,F	b,c 7.7 ± 0.3 B,C,D,E,F	a,b 8.1 ± 0.3 B,C,D,E,F	a,b 8.3 ± 0.3 A,B,C,D,E,F	c 7.6 ± 0.3 B,C,D,E,F	a,b 8.2 ± 0.3 A,B,C,D,E,F	*a* 7.5 ± 0.3	*b* 5.1 ± 0.3	*c,d* 4.0 ± 0.3	*a* 6.9 ± 0.2	*b,c* 4.5 ± 0.4	*d* 3.9 ± 0.5
CNC	4.7 ± 0.4	a 5.2 ± 0.3 B,C,E,F	a,b,c 4.8 ± 0.4 A,B,CD,E,F	b,c 4.4 ± 0.3 A,B,C,D,E,F	a,b 5.0 ± 0.2 A,B,C,E,F	c 4.2 ± 0.4 A,B,C,D,E,F	c,d 4.0 ± 0.5 A,C,D,E,F	*a* 5.7 ± 0.3	*b* 3.4 ± 0.3	*c* 2.5 ± 0.5	*a* 5.5 ± 0.2	*c* 2.3 ± 0.3	*c* 2.0 ± 0.2
EBC	3.8 ± 0.4	2.0 ± 0.3	<1	<1	1.8 ± 0.2	<1	<1	1.5 ± 0.3	<1	<1	1.2 ± 0.2	<1	<1
*E. coli*	2.0 ± 0.5	<1	<1	<1	<1	<1	<1	<1	<1	<1	<1	<1	<1
*L. m*.	n.d.	n.d.	n.d.	n.d.	n.d.	n.d.	n.d.	n.d.	n.d.	n.d.	n.d.	n.d.	n.d.
*S. aureus*	n.d.	n.d.	n.d.	n.d.	n.d.	n.d.	n.d.	n.d.	n.d.	n.d.	n.d.	n.d.	n.d.

TVC—total viable count; LAB—lactic acid bacteria; CNC—coagulase-negative cocci; EBC—enterobacteria count. *E. coli*—*Escherichia coli*; *L. m*.—*Listeria monocytogenes*; *S. aureus*—*Staphylococcus aureus*; n.d.—not determined. * Different small letter(s) in a row indicate a significant difference between DFS subgroups prepared without starter culture (*p* < 0.05). ** Each subgroup of DFS prepared with starter culture is marked by a different capital letter (S-N-H = A; S-N+47 = B; S-N+53 = C; S+N+H = D; S+N+47 = E; S+N+53 = F). Capital letter(s) indicate a significant difference between subgroups of DFS prepared with starter culture and their artisanal subgroup counterparts (*p* < 0.05). *** Different lower-case italicized letter(s) in the same row indicate significant differences between DFS subgroups prepared with starter culture (*p* < 0.05). For abbreviations of subgroups of sausages, see Figure 1.

**Table 3:** *p*-values from three-way factorial ANOVA for the effects of Nitrite, Starter, and Pasteurization on microbial counts (TVC, LAB, CNC, EBC).

Factor/Interaction	TVC	LAB	CNC	EBC
Nitrite	0.0000	0.0021	0.0000	0.0517
Starter	0.0000	0.0000	0.0000	0.0000
Pasteurization	0.0000	0.0000	0.0000	0.0000
Nitrite × Starter	0.0000	0.0226	0.1202	0.5527
Nitrite × Pasteurization	0.0156	0.0375	0.0140	0.0247
Starter × Pasteurization	0.0000	0.0000	0.0000	0.0000
Nitrite × Starter × Pasteurization	0.0435	0.8312	0.3548	0.7016

**Table 4:** Biogenic amines in Sremska sausages. Results belong to three independent repetitions and are expressed as mean values ± standard deviation.

DFS Subgroups	PUT	CAD	HIS	TYM	TRY	PEA	SPD	SPM	Total
A-N-H	a * 129.3 ± 8.5 A,B,C,D,E,F **	a 24.5 ± 2.9 A,B,C,D	a 4.6 ± 0.4 A,B,C	a,b 90.7 ± 4.0 A,B,C,D,E	a 9.5 ± 1.1	<0.25	a 9.0 ± 0.6 A,B,C,D,E,F	a 21.0 ± 2.3 A,B,C,D,E,F	a 288.7 ± 12.9 D,E,F
A-N+47	a 118.8 ± 2.6 A,B,C,D,E,F	a,b 22.8 ± 1.8 A,B,C,D	a 4.6 ± 0.8 A,B,C	a 94.5 ± 3.3 A,B,C	a 9.6 ± 1.2	<0.25	a 9.4 ± 0.8 A,B,C,D,E,F	a 22.7 ± 2.4 A,C,D,E,F	a 282.4 ± 5.0 D,E,F
A-N+53	a 122.8 ± 6.6 A,B,C,D,E,F	a,b 22.1 ± 2.7 A,B,C,D	a 4.9 ± 0.7 A,B,C	b 84.3 ± 3.3 A,B,C,D,E,F	a 10.4 ± 1.1	<0.25	a 8.9 ± 0.7 A,B,C,D,E,F	a 20.5 ± 2.7 A,B,C,D,E,F	a 274 ± 10.7 A,D,E,F
A+N-H	b 83.3 ± 7.2 A,B,C,D,E,F	b,c 15.2 ± 2.1 A,B,C,D,E	a 4.4 ± 0.6 A,B,C	b,c 80.8 ± 2.8 A,B,C,D,E,F	a 9.1 ± 0.9	<0.25	a 9.4 ± 0.8 A,B,C,D,E,F	a 22.9 ± 2.7 C,D,E,F	b 225.2 ± 6.1 A,B,C,F
A+N+47	b 80.6 ± 9.7 A,B,C,D,E,F	b,c 14.4 ± 2.0 A,B,C,D,E	a 5.0 ± 0.8 A,B,C	c 72.3 ± 3.4 A,B,C,D,E,F	a 9.6 ± 1.2	<0.25	a 8.6 ± 0.6 A,B,C,D,E,F	a 17.1 ± 1.7 A,B,C,D,E,F	b 207.7 ± 7.2 A,B,C
A+N+53	b 79.7 ± 3.9 A,B,C,D,E,F	c 13.5 ± 1.9 A,B,C,D,E	a 5.2 ± 0.6 A,B,C	c 71.9 ± 6.8 A,B,C,D,E,F	a 8.4 ± 0.8	<0.25	a 10.0 ± 1.3 A,B,C,D,E,F	a 24.1 ± 2.8 C,D,F	b 212.7 ± 14.8 A,B,C
S-N-H ^A^	*a* *** 58.8 ± 5.7	*a* 58.0 ± 8.6	*a* 19.1 ± 7.6	*a,b* 132.6 ± 6.4	*a* 10.7 ± 1.7	<0.25	*a* 5.7 ± 2.7	*a* 27.2 ± 1.6	*a* 312.2 ± 55.7
S-N+47 ^B^	*a* 55.5 ±7.5	*a* 51.5 ± 6.4	*a* 13.2 ± 5.4	*a* 130.7 ± 4.1	*a* 7.5 ± 2.2	<0.25	*a* 4.9 ± 0.2	*a* 26.1 ± 1.2	*a* 289.4 ± 30.1
S-N+53 ^C^	*b* 44.5 ± 9.7	*b* 41.2 ± 8.4	*a* 13.3 ± 6.1	*b* 141.9 ± 2.7	*a* 8.3 ± 2.2	<0.25	*a* 4.6 ± 0.2	*a* 28.7 ± 3.7	*a* 282.3 ± 19.5
S+N-H ^D^	*b* 40.4 ± 5.0	*b* 38.5 ± 5.8	*b* 5.4 ± 0.5	*c* 108 ± 2.5	*a* 11.0 ± 3.9	<0.25	*a* 5.1 ± 0.3	*a* 29.6 ± 1.2	*b* 237.9 ± 14.6
S+N+47 ^E^	*c* 30.9 ± 1.8	*c* 28.9 ± 1.4	*b* 5.3 ± 1.5	*c* 104.1± 5.7	*a* 6.0 ± 0.7	<0.25	*a* 4.9 ± 0.2	*a* 27.9 ± 1.6	*b,c* 209.7 ± 9.2
S+N+53 ^F^	*c* 20.2 ± 5.4	*c* 21.2 ± 4.1	*b* 3.4 ± 1.4	*c* 99.6 ± 9.9	*a* 11.8 ± 9.0	<0.25	*a* 5.3 ± 0.4	*a* 29.6 ± 1.6	*c* 189.4 ± 26.5

PUT—putrescine; CAD—cadaverine; HIS—histamine; TYM—tyramine; TRY—tryptamine; PEA—phenylethylamine; SPD—spermidine; SPM—spermine. * Different lower-case letter(s) in the same column indicate significant differences between DFS subgroups prepared without starter culture (*p* < 0.05). ** Each subgroup of DFS prepared with starter culture is marked by a different capital letter (S-N-H = A; S-N+47 = B; S-N+53 = C; S+N+H = D; S+N+47 = E; S+N+53 = F). Capital letter(s) indicate significant differences between subgroups of DFS prepared with starter culture and their artisanal counterparts (*p* < 0.05). *** Different lower-case italicized letter(s) in the same column indicate significant differences between DFS subgroups prepared with starter culture (*p* < 0.05). For abbreviations of subgroups of sausages, see Figure 1.

**Table 5:** Pearson’s linear correlation coefficients between microbiological properties and biogenic amine levels of the products.

	Putrescine	Cadaverine	Histamine	Tyramine	Tryptamine	Spermidine	Spermine	Total Biogenic Amines
TVC	0.743	−0.209	−0.481	−0.481	0.205	0.710	−0.580	0.263
LAB	0.777	−0.339	−0.204	−0.599	0.194	0.764	−0.624	0.172
CNC	0.533	0.065	0.064	−0.203	0.213	0.447	−0.323	0.378
EBC	0.221	0.132	0.069	−0.007	0.117	0.140	−0.029	0.253

Abbreviations: TVC—total viable count; LAB—lactic acid bacteria; CNC—coagulase-negative cocci and EBC—enterobacteria count.

**Table 6:** TBARS (malondialdehyde) values (mg/kg) of Sremska sausages. Results are the mean of three independent repetitions and are expressed as mean values ± standard deviation.

Sausages Without Starter Culture *		Sausages with Starter Culture **	
A-N-H	0.14 ± 0.03 A,B,C,D,E,F ***	S-N-H ^A^	0.23 ± 0.02
A-N+47	0.15 ± 0.03 A,B,C,D,E,F	S-N+47 ^B^	0.24 ± 0.02
A-N+53	0.15 ± 0.03 A,B,C,D,E,F	S-N+53 ^C^	0.2 ± 0.03
A+N-H	0.16 ± 0.02 A,B,D,E,F	S+N-H ^D^	0.25 ± 0.04
A+N+47	0.15 ± 0.03 A,B,D,E,F	S+N+47 ^E^	0.25 ± 0.02
A+N+53	0.16 ± 0.03 A,B,D,E,F	S+N+53 ^F^	0.23 ± 0.03

* No significant differences were determined between DFS subgroups prepared without starter culture. ** No significant differences were determined between DFS subgroups prepared with starter culture. *** Each subgroup of DFS prepared with starter culture is marked by a different capital letter (S-N-H = A; S-N+47 = B; S-N+53 = C; S+N+H = D; S+N+47 = E; S+N+53 = F). Capital letters indicate significant differences between subgroups of DFS prepared with starter culture and their artisanal counterparts (*p* < 0.05). For abbreviations of subgroups of sausages, see Figure 1.

**Table 7:** Sensorial attributes of Sremska sausages. Results are expressed as mean values ± standard deviation (the range of values used by the evaluators is 1–7 (1 = dislike extremely, 7 = like extremely)).

DSF	External Appearance	Cross-Cut Surface Appearance	Color	Texture	Juiciness	Odor and Flavor	Overall Acceptability
A-N-H	a * 7.0 ± 0.0 A,B,C,D,E,F **	a 6.9 ± 0.2 A,B,C,D,E,F	a 6.5 ± 0.2	a 5.9 ± 0.9	a 5.4 ± 0.4 A,B,C,D,E,F	a 5.2 ± 0.2 A,B,C,D,E,F	a 5.3 ± 0.5 A,B,C,D,E,F
A-N+47	a 7.0 ± 0.0 A,B,C,D,E,F	b 6.5 ± 0.3 A,B,C	a 6.5 ± 0.2	a,b 6.2 ± 0.5	a,b 5.6 ± 0.5 A,B,C,D,E,F	b 5.7 ± 0.6 A,B,C,D,E,F	b 5.8 ± 0.4 A,D,E
A-N+53	a 7.0 ± 0.0 A,B,C,D,E,F	a 6.9 ± 0.2 A,B,C,D,E,F	a,b 6.6 ± 0.3	a,b,c 6.4 ± 0.4	c 6.2 ± 0.6	c 6.4 ± 0.4	c 6.4 ± 0.4
A+N-H	a 7.0 ± 0.0 A,B,C,D,E,F	a 7.0 ± 0.0 A,B,C,D,E,F	b 6.9 ± 0.2 A,B,C,D,E,F	a,b,c 6.4 ± 0.5	b,c 6.0 ± 0.3	b 5.7 ± 0.3 A,B,C,D,E,F	b 5.9 ± 0.3
A+N+47	a 7.0 ± 0.0 A,B,C,D,E,F	a 6.9 ± 0.2 A,B,C,D,E,F	b 6.9 ± 0.2 A,B,C,D,E,F	c 6.8 ± 0.3 B,C	d 6.8 ± 0.3 A,B,C,D,E,F	d 6.9 ± 0.2 A,B,C,D,E,F	c 7.0 ± 0.0 A,B,C,D,E,F
A+N+53	a 7.0 ± 0.0 A,B,C,D,E,F	a 6.9 ± 0.2 A,B,C,D,E,F	b 6.9 ± 0.2 A,B,C,D,E,F	b,c 6.6 ± 0.4	c 6.2 ± 0.4	a,b 5.5 ± 0.4 A,B,C,D,E,F	b 5.9 ± 0.4
S-N-H ^A^	6.3 ± 0.2 ***	6.2 ± 0.3	6.3 ± 0.2	6.3 ± 0.3	6.3 ± 0.3	6.3 ± 0.2	6.3 ± 0.2
S-N+47 ^B^	6.3 ± 0.3	6.2 ± 0.3	6.3 ± 0.3	6.2 ± 0.3	6.1 ± 0.2	6.2 ± 0.2	6.2 ± 0.3
S-N+53 ^C^	6.3 ± 0.3	6.1 ± 0.2	6.3 ± 0.3	6.1 ± 0.2	6.1 ± 0.2	6.1 ± 0.2	6.2 ± 0.3
S+N-H ^D^	6.3 ± 0.3	6.2 ± 0.3	6.3 ± 0.2	6.3 ± 0.2	6.3 ± 0.3	6.4 ± 0.2	6.3 ± 0.2
S+N+47 ^E^	6.3 ± 0.3	6.3 ± 0.3	6.3 ± 0.3	6.3 ± 0.3	6.2 ± 0.3	6.2 ± 0.3	6.3 ± 0.3
S+N+53 ^F^	6.3 ± 0.3	6.3 ± 0.3	6.3 ± 0.3	6.2 ± 0.3	6.1 ± 0.2	6.2 ± 0.3	6.2 ± 0.3

* Different lower-case letter(s) in the same column indicate significant differences between DFS subgroups prepared without starter culture (*p* < 0.05). ** Each subgroup of DFS prepared with starter culture is marked by a different capital letter (S-N-H = A; S-N+47 = B; S-N+53 = C; S+N+H = D; S+N+47 = E; S+N+53 = F). Capital letter(s) indicate significant differences between subgroups of DFS prepared with starter culture and their artisanal counterparts (*p* < 0.05). *** No significant differences were determined between DFS subgroups prepared with starter culture based on any of the sensorial attributes examined. For abbreviations of subgroups of sausages, see Figure 1.

**Table 8:** Pearson’s linear correlation coefficient between TBARS (malondialdehyde) levels and sensorial attributes of the products.

	External Appearance	Cross-Cut surface Appearance	Color	Texture	Juiciness	Odor and Flavor	Overall Acceptability
TBARS (malondialdehyde)	−0.202	−0.448	−0.488	−0.158	0.386	0.035	−0.092

## Data Availability

The original contributions presented in this study are included in the article. Further inquiries can be directed to the corresponding author.

## Abbreviations

The following abbreviations are used in this manuscript:

CFU	colony-forming unit
CNC	Coagulase-negative cocci
DFS	dry fermented sausages
EBC	Enterobacteria counts
ECC	*Escherichia coli* counts
LAB	Lactic acid bacteria
MDA	malondialdehyde
MRD	maximum recovery diluent
MRS	De Man Rogosa Sharpe broth
TBARS	thiobarbituric acid reactive substances
TVC	total viable counts
